# Real-Time Vehicle Classification System Using a Single Magnetometer

**DOI:** 10.3390/s22239299

**Published:** 2022-11-29

**Authors:** Peter Sarcevic, Szilveszter Pletl, Akos Odry

**Affiliations:** 1 Department of Mechatronics and Automation, Faculty of Engineering, University of Szeged, Moszkvai Krt. 9, 6725 Szeged, Hungary; 2 Department of Technical Informatics, Faculty of Science and Informatics, University of Szeged, Arpad Ter 2, 6720 Szeged, Hungary

**Keywords:** feature extraction, magnetic sensor, time-domain analysis, vehicle classification, vehicle detection

## Abstract

Vehicle count and classification data are very important inputs for intelligent transportation systems (ITS). Magnetic sensor-based technology provides a very promising solution for the measurement of different traffic parameters. In this work, a novel, real-time vehicle detection and classification system is presented using a single magnetometer. The detection, feature extraction, and classification are performed online, so there is no need for external equipment to conduct the necessary computation. Data acquisition was performed in a real environment using a unit installed into the surface of the pavement. A very large number of samples were collected containing measurements of various vehicle classes, which were applied for the training and the validation of the proposed algorithm. To explore the capabilities of magnetometers, nine defined vehicle classes were applied, which is much higher than in relevant methods. The classification is performed using three-layer feedforward artificial neural networks (ANN). Only time-domain analysis was performed on the waveforms using multiple novel feature extraction approaches. The applied time-domain features require low computation and memory resources, which enables easier implementation and real-time operation. Various combinations of used sensor axes were also examined to reduce the size of the classifier and to increase efficiency. The effect of the detection length, which is a widely used feature, but also speed-dependent, on the proposed system was also investigated to explore the suitability of the applied feature set. The results show that the highest achieved classification efficiencies on unknown samples are 74.67% with, and 73.73% without applying the detection length in the feature set.

## 1. Introduction

Automatic vehicle detection technologies provide presence detection, traffic counting, speed monitoring, vehicle classification, and weigh-in-motion data. Thus, they constitute a very important part of intelligent transportation systems (ITS). Vehicle count and classification data are very important during traffic modelling, transportation planning, pavement design, traffic control, and emission/pollution estimation. Vehicle detection systems are also commonly used in parking lots and toll systems.

Vehicle detection systems can be classified into intrusive and non-intrusive technologies [1]. Intrusive detection systems are mounted directly into the pavement surface, in saw-cuts or holes in the road surface, or by tunneling under the surface, and they include inductive loops, piezoelectric detectors, and pneumatic road tubes. Non-intrusive technologies are installed above the roads or on the side of a roadway and include microwave radar, ultrasonic, passive acoustic, active and passive infrared, and video image processing (VIP)-based technologies. Some of these systems are able to monitor multiple lanes. Magnetic sensor-based units can be mounted into the pavement surface or on the side of the roads, so they can be included in both technologies.

Cameras and inductive loops are the most commonly used systems. The advantages of VIP technologies are that they are easily configurable and they can monitor multiple lanes, but their performance is affected by many factors, such as vehicle shadows, day to night transition, fog, rain, snow, etc., and their installation and maintenance are costly. Inductive loop-based systems detect vehicles when they are passing above the sensor based on their metallic content [2]. Speed measurement, vehicle classification, and heading direction estimation in these technologies can be achieved by applying two loops. Vehicle classification is usually realized using the estimated vehicle length and axle-count. Vehicle length is computed using the detection length and the measured speed [3], while the number of axles is estimated by analyzing the waveforms. Some methods were also proposed which apply only a single loop for vehicle classification [4,5]. The main disadvantages of inductive loops are that their mounting and maintenance requires lane closure, their installation requires pavement cutting, and deformations of the road surface largely affect their performance.

Magnetic sensor-based vehicle detection systems are based on the measured changes in the Earth’s magnetic field and can be used as an alternative technology to inductive loops. Similar to inductive loops, they are immune to environmental factors, such as rain, snow, or fog. The main advantages of these systems compared to loops are that they require less pavement cut, and they are less susceptible to stresses of traffic. The use of batteries can further decrease the pavement damage and the installation cost, because there is no need to install cables to the detector. These models can form wireless sensor networks (WSNs) [1,6,7]. Magnetometer-based vehicle detection systems can also effectively work when they are mounted on the side of the roads [8,9].

Another advantage of the magnetic sensor-based technology is that even a single sensor unit can effectively estimate the heading direction, which can be used for detecting vehicles driving in the wrong lane, e.g., while overtaking. This can be achieved by analyzing the measurements in the axis pointing in the direction of movement, or for more precise results, by using a two-axis sensor, where the axes are parallel with the ground [10,11,12].

Classification of vehicles using the magnetic sensor technology can be done using two sensors by measuring vehicle length and/or by processing the magnetic signature of the vehicle, which is much more detailed than an inductive loop-based signature. Counter to the inductive loops, vehicle axles cannot be detected so easily in the waveforms.

Magnetic sensors can be used fused together with other sensor types. Accelerometers mounted on the pavement surface can measure vibrations caused by vehicles passing near the sensor. The use of accelerometers alone was also reported in the literature [13], but they were mainly utilized together with magnetic sensors [14,15,16,17]. In these systems, accelerometer data are applied to identify axle locations using peak detection algorithms, while the magnetometers are used to determine vehicle arrival, departure, and speed.

Magnetometer measurements are affected by temperature drifts, which is a complicating factor in traffic monitoring systems, due to the many changes in the temperature of the pavement [1]. This effect can make parking occupancy detection very hard, since the vehicles are standing above the sensor for a long time, but with proper algorithms these systems can be effectively used for this purpose too [18,19,20].

This paper studies the online classification of vehicles into multiple classes using measurements from only a single magnetic sensor mounted into the surface of the pavement.

The rest of the paper is organized as follows. Section 2 presents related research results and the contributions of the proposed work. The measurement system and the data acquisition are presented in Section 3. The proposed classification algorithm is described in Section 4. The experimental results are discussed in Section 5, while Section 6 summarizes the results of the paper.

## 2. Related Work and Contributions

### 2.1. Vehicle Detection Using Magnetic Sensors

Various algorithms exist to detect vehicle presence using magnetometer-based systems, and reported results show that almost 100% detection efficiency can be achieved. Both in-pavement and roadside technologies were applied in these works. In the case of in-pavement systems, the incorrect detections are mainly caused by vehicles passing in the neighboring lanes. Most of the algorithms apply fixed or adaptive thresholds. The problem with fixed thresholds is that they cannot deal with the variations of the environment. Adaptive thresholds solve this difficulty by following the drifts during time. Many algorithms require a sequence of measurements to exceed the range defined by the thresholds to declare the arrival or the departure of the vehicle, since this can filter some false detections. Some authors applied only one sensor axis in their algorithm [21,22,23], which proved to be sufficient for efficient vehicle detection. Other works utilized multiple axes in the detection algorithm [1,24]. Beside the measurement values on the sensor axes, the computed magnetic field magnitude [6,24,25,26,27,28] and the magnetic field angle between sensor axes [29] can be also effectively used for detection purposes. Threshold-based algorithms were used in [6,25,29,30,31], while the authors of [26,27] utilized signal variances.

### 2.2. Vehicle Classification Using Magnetic Sensors

The waveforms depend on the vehicle’s position from the sensor and exhibit a large variability even among vehicles of the same class. Thus, the classification of vehicles based on magnetic signatures is a very challenging task [32].

Many works applied two or more measurement units [21,24,33,34,35], which enables the computation of vehicle speed and length. These methods mostly rely on measured vehicle length during classification.

Other approaches utilize only a single unit, which can decrease the cost of the system. Table 1 summarizes the placement of the sensors, the number of applied vehicle classes and samples, the used classification methods, and the achieved efficiencies in related research dealing with vehicle classification utilizing only a single unit, while Table 2 shows the used data types and features during feature extraction.

As can be seen in Table 1, both roadside [10,26,36,37,38,39] and in-pavement [1,25,27,40,41,42,43] installation were considered in relevant works, and even the usage of a unit fixed atop of the roadway was proposed in [32]. The proposed methods in [42,43] utilized single units with two magnetic sensors, which were used to estimate vehicle speed and length. The other methods determine the class of the passing vehicle by only using different features extracted from the time-series.

As shown in Table 2, mostly the waveforms measured on one or more sensor axes were applied during feature extraction (the orientation of the *X*, *Y*, and *Z* axes is the same as described in Section 3.2), but the magnetic field magnitude (*F*) was also widely used. The methods mostly utilized various time- and frequency-domain features. In [32], principal component analysis (PCA)-based dimension reduction was used on fast Fourier transform (FFT)-based components to construct the applied feature set. In [38], mel frequency cepstral coefficients (MFCC) were extracted from the magnetic signals after the discrete Fourier transform (DFT) was performed. Feng et al. applied histogram of oriented gradients (HOG) features extracted from the images containing the waveforms [41]. In [39], such images were directly used as the inputs on the convolutional neural network (CNN). In related research, vehicles were classified into 2–5 classes, but most of the works applied a very small number of samples for classification. Most algorithms utilized the classification trees (CT) and the support vector machines (SVM) for classification, but the k-nearest neighbor (k-NN) algorithm, the vector quantization (VQ), and the direct hill-pattern matching classifier were also applied. In [37], four classifiers, i.e., the CT, the SVM, the k-NN, and the random forest (RF), were compared, of which the SVM provided the highest classification accuracies. In [38], the dynamic time warping (DTW) was utilized to improve the selection of training samples.

### 2.3. Motivation and Contributions

The goal of this research was to develop an online vehicle classification method utilizing a single magnetometer-based unit. A unit mounted into the pavement surface was used, because the measurement signals are more detailed and carry more information than when the sensor is installed on the side of the roads (described in Section 3.1). The contributions of the work can be summarized as follows:

- In this study, nine vehicle classes were defined and utilized to explore the capabilities of the magnetometer-based technology. This number is much higher compared to relevant methods, where, as described in Section 2.2, vehicles were classified into two to five classes.
- Drawbacks of reported studies are, that most works applied only a small number of collected samples, and many do not consider unknown data to validate their developed algorithms. In this work, a very high number of samples were used to construct training and validation datasets.
- Related works mainly do not deal with the implementation of the algorithms. In the proposed system, all parts of the vehicle classification algorithm are realized on the used microcontroller-based hardware. To achieve online and real-time operation, the feature set consists of only time-domain features, which require less computation and memory resources than necessary for frequency-domain analysis, which were also widely used in other studies.
- Different novel feature extraction modes are proposed to minimize the number of used features and the possible cost of the system. Various combinations of applied sensor axes were also compared using the proposed feature set to find the optimal setup.
- Other disadvantages of many works include the utilization of the length of the detection as one of the inputs in the classification stage. This feature provides valuable information about the length of the vehicle, but it obviously has a negative effect on recognition efficiency if the unit is placed in a location where the speed is different to the location where the training samples were collected. The effect of this feature in the proposed system was also investigated to explore the suitability of the applied feature set.

Previously, an initial investigation with a smaller feature set was presented in [44], where six vehicle classes were used: motorcycles, cars, vans, trucks, buses, and other. The highest recognition rate was 88.44% on training samples and 70.83% on validation data. The main results proposed in this study are based on [45].

## 3. Measurement

Magnetometers are passive sensors that measure the strength of the Earth’s magnetic field at a given point. Metallic objects such as vehicles cause local distortions, which can be measured by these sensors. Vehicle presence can be determined based on these distortions. Vehicles have different magnetic fingerprints due to their different ferromagnetic composition.

Magnetic sensor-based systems, as described earlier, can be installed both in the pavement surface and on the roadside, but the distortions are much stronger when the sensors are mounted below the passing vehicles. The signals are also more uniform in the case of roadside sensors, since many different ferromagnetic parts pass near the sensors when they are mounted into the pavement [21]. Another disadvantage of roadside installation is that these units can only detect vehicles in the adjacent lane. Vehicles with high metallic content passing in the neighboring lane can cause false detections in the case of in-pavement mounting. Using a three-axis magnetometer, the axis pointing to the neighboring lane can be utilized to filter these false detections [44,46].

### 3.1. Measurement Unit

The used hardware is based on a Honeywell HMC5843 magnetometer, which is a small (4 mm × 4 mm × 1.3 mm), surface mount, multi-chip module designed for low field magnetic sensing in three axes. The sensor utilizes anisotropic magnetoresistive (AMR) technology and features precision in-axis sensitivity and linearity, and very low cross-axis sensitivity. The ASIC also contains an I2C serial bus interface. It can measure up to ±650 μT in 12-bit resolution, and the highest sampling frequency is 50 Hz.

The unit also contains a microcontroller, which is used to set up the sensor, to read the measurements via an I2C interface, and to forward the data via RS-485 interface to a central unit, which is a PC. The supply and the communication are realized using a cable.

### 3.2. Data Acquisition

A single measurement unit was installed into the pavement surface in a plastic box for data acquisition. The chosen location was between two intersections (150–200 m from both) on one of the main roads of Subotica, Serbia. The vehicles were moving with nearly constant speed when they were passing above the sensor, since they had enough time from the intersection to accelerate and reach their desired speed. The speed limit is 50 km/h, but the speed of the passing vehicles varied greatly, and many drivers even exceeded the limitation. The location was very advantageous, since it is a frequented road with various vehicle classes passing.

**Figure 1:**
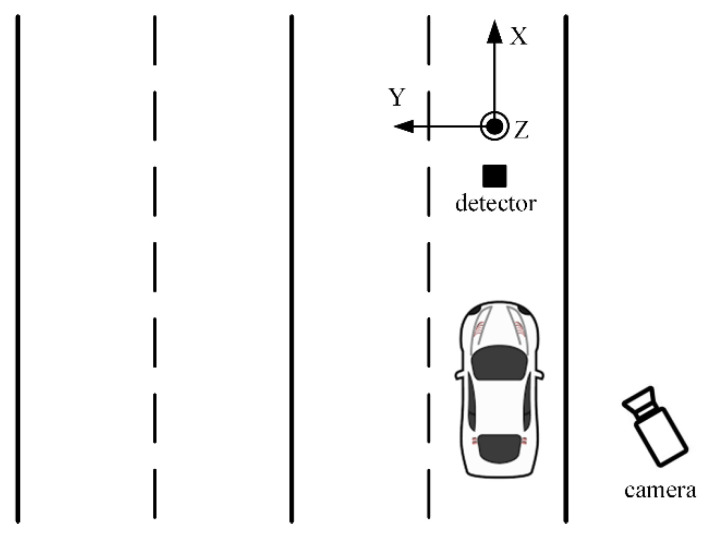
Sensor placement for data acquisition.

In-pavement mounting was chosen, since the measurement signals are more detailed and carry more information than when the sensors are mounted on the side of the roads. The unit was mounted into the middle of the outer lane, as shown in Figure 1. The sensor’s *X* axis pointed in the movement direction, the *Y* axis pointed to the neighboring lane, and *Z* was orthogonal with the pavement surface. The sensor unit was installed into the pavement, 5 cm beneath the surface.

The developed vehicle detection algorithm (described in Section 4) was implemented on the installed measurement unit. The applied sampling frequency was 50 Hz. The software of the device sent the measurement data and the value of the detection flag to a central unit via the RS-485 interface. Data acquisition software on the server side received and stored the measurement data. To validate the detections, and to determine the class for each recorded sample, a camera was installed beside the road. The data acquisition software saved camera images at every falling edge of the detection flag.

**Figure 2:**
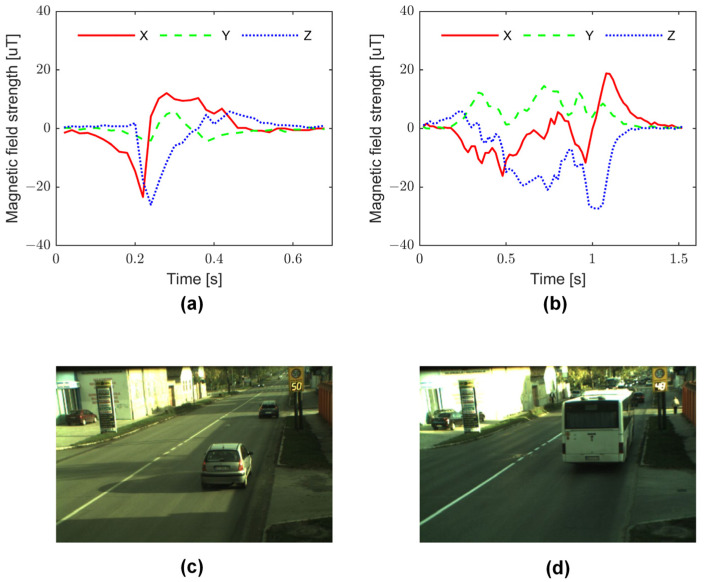
Measured signals on the three sensor axes when a car (**a**) and a bus (**b**) are passing above the sensor, and the corresponding images of the vehicles (**c**,**d**).

Data acquisition was performed during multiple months in various weather conditions, and altogether more than 30,000 samples were collected. Figure 2 shows the measurement signals on the three axes and the corresponding vehicle images when a car and a bus pass above the sensor. It can be seen in Figure 2 that the signals generated by a bus are much more complex than the disturbances caused by a passing car, i.e., it has more direction changes, local minima/maxima, zero crossings, etc.

## 4. Vehicle Classification Algorithm

The used vehicle classification algorithm consists of three main parts: vehicle detection algorithm, feature extraction, and classification. To perform real-time and online vehicle detection and classification, all parts should be easily implementable on the microcontroller of the used hardware.

**Figure 3:**
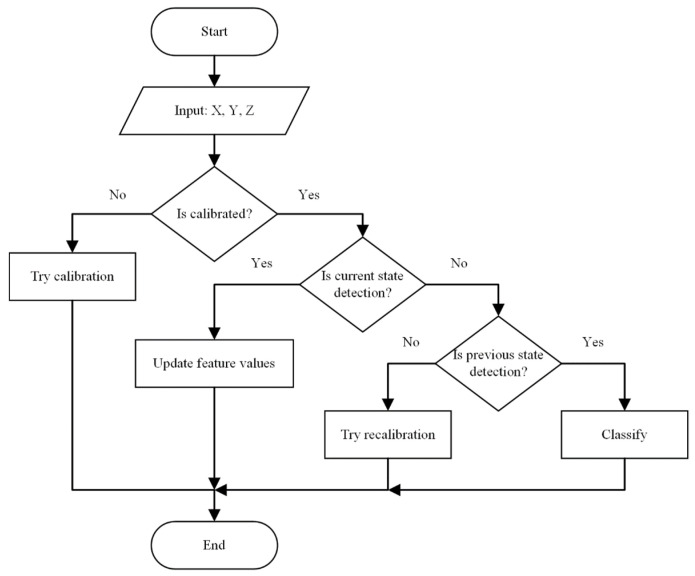
The flowchart of the proposed vehicle classification algorithm.

The flow chart of the vehicle classification algorithm for one measurement cycle is shown in Figure 3.

### 4.1. Vehicle Detection Algorithm

Vehicle detection is a very important part of the classification process, since it should follow the environmental changes, and act the same way for different vehicles. These environmental changes cause slow variations in the offsets on the three sensor axes.

The used algorithm was designed and optimized for classification applications and was previously proposed in [44]. The basic parts of the algorithm are the following:

- When the unit is turned on, the calibration process is run. This process uses a calibration range size, which must be slightly larger than the peak-to-peak noise level, because it should follow the slow changes in the environment. This noise level should be estimated after the installation of the unit in the current location, when no vehicles are near to the sensor. During calibration, the highest and lowest measurement values are monitored in a measurement window for all three axes. The difference between the highest and lowest values must be smaller than the calibration range size. The calibration is restarted if the difference exceeds the range size at any time in the window at any of the axes. This filters the factors which affect the magnetic field near the sensor. The size of the measurement window should be at least 1 s based on experience. If the calibration is successful, the upper and lower calibration and detection thresholds are calculated for all axes. This is done by equally stretching the range determined by the highest and lowest values to the width defined by the range sizes.
- Vehicle presence is declared if the measurement values exceed the detection threshold at both *X* and *Z* axes.
- The detection flag is cleared if the measurement values on both *X* and *Z* axes are between the calibration thresholds for a previously defined number of measurements. The algorithm should be suitable for detecting vehicles with trailers, so the used length should be calculated using the potential speed on the location and the possible distance between the vehicle and the trailer.
- A recalibration is attempted always when vehicle presence is not declared. The process is the same as during calibration, but the measured highest and lowest measurements must fit into the previous calibration range. This enables the following of the environmental changes.

The overall efficiency of the detection algorithm is 94.15% [44]. The algorithm detects all vehicles passing above the sensor, and only motorcycles can cause failures if they are not passing near to the detector. False detections cause the rest of the failures. These are generated by vehicles with high metallic content passing in the neighboring lane. The detection range size is set to be as small as possible to achieve fast reaction when the vehicles are approaching the sensor. This enables the processing of the entire magnetic signature of the vehicle in the feature extraction stage. The number of false detections could be decreased with raising the thresholds, but this could also lower the detection rate of motorcycles, and important parts of the magnetic signature could also be lost. Approximately 97% of the false detections can be filtered out when the detection flag is cleared using a properly set up rule-based algorithm [46], which can further improve the efficiency of the classification process.

### 4.2. Feature Extraction

The inputs in the classification stage are formed by the extracted features on the time-series data. Features are extracted in the detection window, but the last measurements, which are used for clearing the detection flag, are not utilized. The chosen feature types are based on only time domain analysis because they need little computation, and they also do not require the storage of all measurements in the window. This speeds up the processing time and decreases the necessary memory. The feature values are updated after every new measurement, so they can be immediately passed to the classifier when the detection is cleared.

#### 4.2.1. Feature Types

Feature types were chosen based on their potential ability to detect vehicle axles and overall metallic content. It is also important that the features should be immune to speed variations. Some features were previously not applied in related works for vehicle classification purposes but are very popular in other pattern recognition applications, such as human movement classification [47]. The used feature types were the following:

- Detection length (DL): The number of measurements in the detection window.
- Highest value (MAX) and lowest value (MIN): The highest and lowest measurement values in the detection window.
- Place of the highest and lowest values (PlaceMax, PlaceMin): The indexes of the measurements where the MAX and MIN points were found, both divided with the detection length.
- Range changes (RCH): The calibration thresholds define three ranges in the signal values, one above the upper threshold, one under the lower, and one between them. This feature measures how many times has the signal switched ranges.
- Number of local maxima and minima (NumLocMax, NumLocMin): The number of local maxima in the range above the upper threshold, and the number of local minima in the range under the lower threshold. A point is considered as a local maximum (minimum) if it has local minima (maxima) before and after it, and the differences in the amplitude are higher than the peak-to-peak noise value.
- Mean absolute value (MAV): The mean absolute amplitude value, which can be calculated as
  (1) $$\mathrm{MAV}=\frac{1}{N}\overset{N}{\underset{i=1}{\sum }}\left|x_{i}\right|$$
  where *N* is the number of samples in the detection window and *x_i_* are the signal amplitudes at the given index.
- Mean value (MV): The mean amplitude value.
- Number of slope sign changes (NSSC): The number of direction changes, where among the three consecutive values the first or the last changes are larger than a predefined *th* threshold, which is the peak-to-peak noise level (2).
  (2) $$\mathrm{NSSC}=\overset{N-1}{\underset{i=2}{\sum }}\left[f\left[\left(x_{i}-x_{i-1}\right)\cdot \left(x_{i}-x_{i+1}\right)\right]\right],f\left(x\right)=\left\{\begin{array}{c} 1,\;\mathrm{if}\left(x\geq th\right)\\ 0,\;\mathrm{otherwise} \end{array}\right.$$
- Number of zero crossings (NZC): The number of times when the amplitude values cross the zero-amplitude level and the difference between the values with opposite signs is larger than the threshold:
  (3) $$\mathrm{NZC}=\overset{N-1}{\underset{i=1}{\sum }}\left[\mathrm{sgn}\left(x_{i}\cdot x_{i+1}\right)\cap \left|x_{i}-x_{i+1}\right|\geq th\right],\;\mathrm{sgn}\left(x\right)=\left\{\begin{array}{c} 1,\;if\left(x\geq 0\right)\\ 0,\;otherwise \end{array}\right..$$
- Average waveform length (AWL): The length of the waveform over the detection window divided by the number of samples in the window:
  (4) $$\mathrm{AWL}=\frac{1}{\mathrm{DL}}\sum_{i=1}^{\mathrm{DL}-1}\left|x_{i+1}-x_{i}\right|$$
- Root mean square (RMS): The calculation of the RMS can be performed as given in (5).
  (5) $$\mathrm{RMS}=\sqrt{\frac{1}{N}\overset{N}{\underset{i=1}{\sum }}x_{i}^{2}}$$
- Willison amplitude (WAMP): The number of amplitude changes in the window, which are higher than the given threshold level (6).
  (6) $$\mathrm{WAMP}=\overset{N-1}{\underset{i=1}{\sum }}\left[f\left(x_{i}-x_{i+1}\right)\right],\;f\left(x\right)=\left\{\begin{array}{c} 1,\;\mathrm{if}\left(x\geq th\right)\\ 0,\;\mathrm{otherwise} \end{array}\right.$$

#### 4.2.2. Extraction Modes

In related works, the raw signal values on the *X*, *Y*, and *Z* axes and the *F* magnetic field magnitude were utilized for feature computation. In this work, various novel extraction modes were tested, which can possibly increase efficiency and/or decrease the required feature number. Feature extraction is performed using raw sensor measurements and aggregated data, which are computed using the raw measurement data. The tested aggregation modes were motivated by the fact that both the strength and the direction of the magnetic field at the sensor will change when a vehicle passes by. A lower input number in the classification process can decrease the necessary computation time and the required memory space for the implementation of the classifier. The following extraction modes were applied:

- Measurement axes *(X*, *Y*, *Z)*: The features were computed using the raw measurement values on each axis. The calibrated offsets were subtracted from the measured values.
- Absolute values *(X_abs_*, *Y_abs_*, *Z_abs_)*: The computed absolute values were applied on each measurement axis, which were calculated after the offsets were subtracted from the measurement values.
- Magnitude from the origin *(XY_O_*, *XZ_O_*, *YZ_O_*, *XYZ_O_)*: Magnitude values were computed in three dimensions and in two dimensions using different combination of the axes. The magnitude of the calibration point was subtracted from computed magnitudes. These data provide information about the changes in magnitude in different planes and in 3D based on the sensor frame compared to the magnitudes given by the calibration point.
  (7) $$XY_{O}=\sqrt{X^{2}+Y^{2}}-\sqrt{X_{cal}^{2}+Y_{cal}^{2}}$$
  (8) $$XZ_{O}=\sqrt{X^{2}+Z^{2}}-\sqrt{X_{cal}^{2}+Z_{cal}^{2}}$$
  (9) $$YZ_{O}=\sqrt{Y^{2}+Z^{2}}-\sqrt{Y_{cal}^{2}+Z_{cal}^{2}}$$
  (10) $$XYZ_{O}=\sqrt{X^{2}+Y^{2}+Z^{2}}-\sqrt{X_{cal}^{2}+Y_{cal}^{2}+Z_{cal}^{2}}$$
  where *X_cal_*, *Y_cal_*, and *Z_cal_* define the calibration point, which are the middle points between the upper and lower calibration thresholds on each sensor axis.
- Angles *(XY_A_*, *XZ_A_*, *YZ_A_)*: The difference between the angle computed for a measurement point and the angle of the calibration point. The angles were determined for all three planes defined by the sensor axes using (11)–(13).
  (11) $$XY_{A}=\tan^{-1}\left(Y/X\right)-\tan^{-1}\left(Y_{cal}/X_{cal}\right)$$
  (12) $$XZ_{A}=\tan^{-1}\left(Z/X\right)-\tan^{-1}\left(Z_{cal}/X_{cal}\right)$$
  (13) $$YZ_{A}=\tan^{-1}\left(Z/Y\right)-\tan^{-1}\left(Z_{cal}/Y_{cal}\right)$$
- Magnitude from the calibration point *(XY_C_, XZ_C_, YZ_C_, XYZ_C_)*: Magnitudes were computed using the differences between the measurement points and the calibration point. The computation was also conducted for two and three dimensions, as given in (14)–(17).
  (14) $$XY_{C}=\sqrt{{\left(X-X_{cal}\right)}^{2}+{\left(Y-Y_{cal}\right)}^{2}}$$
  (15) $$XZ_{C}=\sqrt{{\left(X-X_{cal}\right)}^{2}+{\left(Z-Z_{cal}\right)}^{2}}$$
  (16) $$YZ_{C}=\sqrt{{\left(Y-Y_{cal}\right)}^{2}+{\left(Z-Z_{cal}\right)}^{2}}$$
  (17) $$XYZ_{C}=\sqrt{{\left(X-X_{cal}\right)}^{2}+{\left(Y-Y_{cal}\right)}^{2}+{\left(Z-Z_{cal}\right)}^{2}}$$

Since the extraction modes based on the absolute values and the magnitudes calculated from the calibration point always provide positive values, the following feature types were not computed in these cases: MIN, PlaceMin, NumLocMin, NZC, and MAV.

### 4.3. Classification

The multilayer perceptron (MLP) neural network was used for classification in the proposed algorithm. Due to its high recognition efficiency and easy implementation on microcontroller-based systems, this classifier proved to be a good solution for online classification purposes compared to other well-known classifiers [47].

Artificial neural networks (ANN) are motivated by biological neural systems and used to approximate target functions. The MLP is a feedforward ANN, where neurons are organized into three or more layers (an input and an output layer with one or more hidden layers), with each layer fully connected to the next one using weighted connections. A neuron has an activation function that maps the sum of its weighted inputs to the output. Usually, ANNs are trained using the backpropagation algorithm, which uses gradient descent to tune network parameters to best fit a training set of input-output pairs.

In this work, three-layer networks were applied. The hyperparameters of the network structure can be seen in Table 3. The number of input neurons was equal to the size of the feature vector, while in the output layer a neuron was assigned to each class. To achieve easy implementation, it is important to minimize the number of neurons in the hidden layer. Various configurations were tested based on the number of hidden layer neurons to find the optimal setup. Hyperbolic tangent sigmoid transfer function was used in the hidden layer, while the neurons in the output layer were created using the linear transfer function. The output neuron with the largest output was assigned as the class of the given sample.

The training of the MLPs was conducted offline. This procedure is time consuming since various configurations must be tested and even one training can require multiple hours, which is much more than in the case of other well-known classifiers [47]. The training time depends on various factors, e.g., the number of neurons in different layers, the number of samples, the hyperparameters used during training, the initial weights, etc. Although it is a complex and time-consuming process to find the optimal network for a given task, it does not affect its real-time operation after implementation.

## 5. Experimental Results

### 5.1. Vehicle Classes

Considering the capabilities of magnetic sensors, nine possibly separable vehicle classes were defined. The vehicle types and the number of axles for each class are shown in Table 4.

Some vehicle classes (e.g., cars) passed frequently at the location. An equal number of samples (130 per class) was utilized for all classes during the training of the MLP networks to obtain better evaluation. The training of the MLPs was done using 80 (61.54%) samples, while the remaining 50 (38.46%) were used as unknown inputs for the validation of the trained classifiers. In the training process, 70% of the training data were used as training inputs and 30% as validation inputs.

### 5.2. Datasets

Since the classification algorithm should be implemented on the microcontroller-based system, it is important to minimize the number of inputs. The waveforms on the *X* and *Z* axes should carry most of the useful information. The *Y* axis can give valuable information about the position of passing vehicle, but it can also be largely affected by vehicles passing in the neighboring lane. The aggregation of the measurement data can further decrease the number of features, and possibly increase the classification efficiency. To find the optimal configuration, it was necessary to test different combinations of used axes. Using the described five feature extraction modes, altogether, 18 different combinations were defined:

- Measurement axes:
  1. *X*, *Y*, and *Z*;
  2. *X*;
  3. *Z*;
  4. *X* and *Z*.
- Absolute values:
  5. *X_abs_*, *Y_abs_*, and *Z_abs_*;
  6. *X_abs_*;
  7. *Z_abs_*;
  8. *X_abs_* and *Z_abs_*.
- Magnitudes from the origin:
  9. *XY_O_*, *XZ_O_*, *YZ_O_*, and *XYZ_O_*;
  10. *XZ_O_*;
  11. *XY_O_*, *XZ_O_*, *YZ_O_*;
  12. *XYZ_O_*.
- Angles:
  13. *XY_A_*, *XZ_A_*, *YZ_A_*;
  14. *XZ_A_*.
- Magnitude from the calibration point:
  15. *XY_C_*, *XZ_C_*, *YZ_C_*, and *XYZ_C_*;
  16. *XZ_C_*;
  17. *XY_C_*, *XZ_C_*, *YZ_C_*;
  18. *XYZ_C_*.

The detection length feature provides vehicle length information, but it depends on the vehicle speed. Since other chosen features should be potentially immune to speed differences, it was reasonable to explore the effect of this feature on classification efficiency. This is important because not using the detection length in the feature set could enable the system to work efficiently on other locations where the potential speed of the vehicles is different. By adding this feature to all defined combinations, altogether, 36 different datasets were tested.

### 5.3. Performance Evaluation

To provide more valid results, five different training and validation sets were generated for all dataset types by randomly distributing the 130 samples. All five datasets for the 36 input combinations were tested with five, 10, 15, 20, 25, and 30 neurons in the hidden layer. For all setups, five sets of training were performed since the training of the neural networks largely depends on the initial weights. In the further comparison, the results with the highest efficiency on validation data from the five training sets were utilized. The training of the MLPs was realized in MATLAB environment. The hyperparameters of the training process are summarized in Table 5.

Table 6 summarizes the required feature numbers and the average classification efficiencies on training and validation samples for each of the 36 datasets. The computation of the average efficiencies was performed using the efficiencies when the best results on unknown samples were obtained for each of the five random sets. It can be observed that the highest efficiencies can be achieved using the feature extraction mode where the magnitudes from the origin were utilized. The highest recognition rate on validation data, 74.67%, was obtained using the magnitude changes in three dimensions (*XYZ_O_*) together with the DL feature. The number of features in this dataset is 15, which is also acceptable compared to other setups. Without applying the information about the length of the detection, the highest result was 73.73%, achieved using features extracted in the *XZ*-plane (*XZ_O_*), which is a significant result taking into account the number of classes and achieved classification efficiencies in related works. The number of features in this dataset is 14. This feature set with the DL provided 74.44%, which is only 0.23% lower than with the *XYZ_O_*-based extraction. This shows that the *Y* axis does not carry any additional information if the *X* and *Z* axes are used. The extraction mode applying the magnitudes from the calibration point requires a smaller number of features compared to the other magnitude-based mode, but the classification efficiencies are also lower. The differences are mostly higher than 3%. Recognition rates between 70% and 72% can be achieved using the features extracted from the waveforms of the measurement axes. Applying only the *X* or the *Z* axis provides very similar results, 71.33% and 71.24% without the detection length, and 72.22% and 72.36% with the DL, respectively. This shows that notable results can be achieved using even a single axis sensor, which can largely decrease the cost of the system. Utilizing the features from the two axes together even decreases the average recognition rate, although it requires double number of features. The average efficiencies are 70.13% without and 71.64% with the DL. The extraction mode utilizing the absolute values requires less features, but the recognition rates are smaller for 1–3% for the same axis combinations. Applying the changes in angles for feature computation can also provide high classification rates. Using only the signals in the *XZ*-plane can result in 71–72% efficiency on validation samples. The highest classification accuracies on training data can be achieved using the datasets with the highest feature numbers. In more datasets, approximately 82–84% can be reached, but the efficiencies on unknown data are smaller in these cases than when the setups with lower feature numbers are utilized. In case of the datasets which provide the best results on unknown data (above 72%), approximately 80–81% efficiency was obtained on training samples. Analyzing the effect of adding the DL feature to the feature vector, it can be stated that it does not increase the classification efficiencies significantly. The recognition rates are increased in average by 1.28% ±1.63% for training data and 0.80% ±1.37% for unknown samples. This shows that the used speed independent feature types are sufficient to extract the necessary information from the waveforms.

The average misclassification rates on the five random validation sets utilizing the *XZ_O_*-based features with and without the DL feature are presented in Table 7. This extraction mode provided the highest results on unknown data. It can be observed that the motorcycles can be recognized with more than 96% in both cases. Cars are correctly classified for 90.0% of the samples with, and 87.6% without the DL feature. Most of the improperly classified samples are assigned as vans (4.8% and 6.8%). Buses can be recognized with misclassification rates of 11.2% and 13.6%. Articulated buses have lower classification rates, and the DL feature even lowers the efficiency. The recognition rates are 76.4% with and 83.2% without the DL. Most of the misclassifications occur as trucks with trailer (8.8% and 6.8%) and buses (11.6% and 5.6%). The most positive effect of the added DL feature can be noticed at vans, which are classified with the highest misclassification rates (44.8% and 52%). Further, 24.0% of the samples are classified as trucks for both datasets, and 7.6% and 8.4% as cars with trailer. The improvements between the two sets can be noticed in cars, since using the DL, the misclassification rates decrease from 17.6% to 10.4%. This result is reasonable, since both classes have two axles and do not differ significantly in metallic content. The rest of the vehicle classes, trucks, trucks with trailer, and tractor trailers, can be recognized with similar rates, around 55–60%. Trucks are mostly misclassified as vans (10.8% and 8.4%) and buses (12.4% and 18.0%), trucks with trailers as tractor trailers (16.8% and 21.6%) and articulated buses (13.2% and 11.6%), while tractor trailers as trucks with trailers (13.6% and 10.8%), buses (12.8% and 10.8%), and articulated buses (8.4% and 9.6%).

The required number of hidden layer neurons for achieving 97% convergence in efficiency using the mean values from the five random sets can be seen in Table 8. Analyzing the results, it can be observed that mostly 10 (58.33%) and 15 (30.56%) neurons are required for validation samples. To reach convergence on training data, more, between 15 and 25 neurons are necessary. This analysis shows that the tested range in the number of hidden layer neurons is sufficient for solving the given task.

### 5.4. Implementation

The implementation of MLPs requires the storage of three parameter types: input ranges, weights, and biases. Input ranges are used for the normalization of input values, and the matrices consist of the highest and lowest values for all inputs. Two weight matrices are required, one connecting the input layer with the hidden layer, and the second connecting the hidden layer and the output layer. The dimensions of the matrices can be given using the sizes of the layers. Bias values are used in all neurons of the hidden and the output layer.

**Figure 4:**
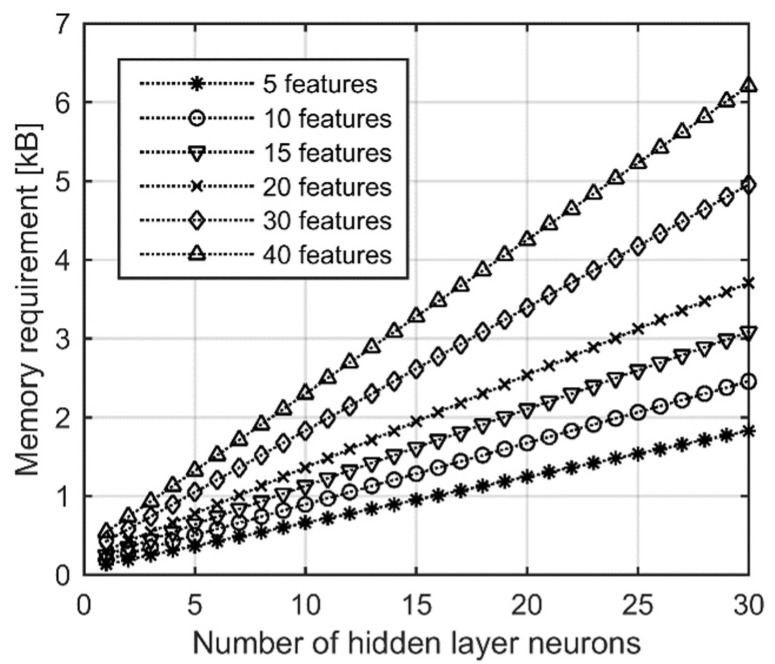
Memory requirements for the implementation of MLP networks.

The necessary memory for different features and hidden layer neuron numbers can be seen in Figure 4. The required memory spaces are given in bytes. Since all parameter types are floating-point numbers, during computation, 4 bytes were counted for each parameter. It can be observed from Figure 4 that even with the largest examined feature numbers and hidden layer neuron numbers, which are 40 and 30, respectively, the required memory for storing the weights of the MLP is approximately 6 kB. This is much lower than the usual memory capacity of microcontrollers, which shows that the classifier is implementable on such embedded systems.

The testing shows that after the detection is cleared, the vehicle class can be determined in 1–2 measurement cycles (20–40 ms), without affecting the other parts of the algorithm. The required time depends on the size of the network.

## 6. Conclusions

In this study, a novel, real-time online vehicle classification system was presented, which utilizes only a single tri-axial magnetic sensor. Data acquisition was performed during multiple months in a real environment using a unit mounted into the pavement. Vehicle detection was performed using an adaptive threshold-based algorithm, which can follow the environmental changes. A camera was also installed beside the road, and images were saved for every detection to determine the vehicle class for each sample.

Nine vehicle classes were defined, and 130 samples per class were used for the training and validation of the classifiers. The utilized features for classification were extracted in the detection windows. The chosen feature types are based on only time domain analysis and have low computation and memory requirements.

Since the classification algorithm should be implemented on the microcontroller-based system, it is important to minimize the applied feature number. Various novel feature extraction modes and used sensor axes were tested to find the optimal configuration. The effect of the detection length was also investigated, since it depends on the speed of the vehicle. Altogether, 36 different datasets were constructed and compared. The classification was performed using three-layer MLP networks.

The obtained results show that using aggregated data in the form of angles or magnitudes can even increase the classification efficiencies besides decreasing the number of inputs. The highest recognition rates on validation data were achieved using magnitudes computed from the origin in the *XZ*-plane, 74.67% with, and 73.73% without applying the detection length. Using only the *X* or the *Z* axis can provide 71–72%, which shows that even a single axis sensor can effectively classify vehicles into multiple classes. The length of the detection on average increases the classification efficiencies only by 1.28% ±1.63% for training data, and 0.80% ±1.37% for unknown samples, which shows that the used features can effectively extract the information from the waveforms. The necessary feature number in the best sets was 14–15, while convergence on unknown samples can be noticed with 10–15 neurons.

The testing shows that the proposed algorithm can be implemented on the microcontroller of the unit, and the vehicle class can be determined in 1–2 measurement cycles (20–40 ms) after the detection is cleared. The solution could also be effectively used in WSN applications, since the classification algorithm runs online, and there is no need to transmit measurement data to a central unit.

The future goals of this research include improving the recognition efficiency, adding more features which could discriminate different trucks and buses, and finding the features with most influence using different feature selection algorithms to reduce the required number of inputs in the classification stage. To deal with the diversity of various locations, data should be collected from further locations and applied together during the training process.

## Figures and Tables

**Table 1:** Summary of relevant works.

Related Work	Sensor Placement	Vehicle Classes	Sample Number	Classifier	Efficiency
[10]	side of the road	3 (heavy tracked vehicle; light tracked vehicle; light wheeled vehicle)	93	SVM	86.27%
[26]	side of the road	5 (motorcycle; hatchback; sedan; SUV; bus)	100	CT	97%
[36]	side of the road	4 (hatchback; sedan; bus; multi-purpose vehicle)	300	k-NN	95.46%
[37]	side of the road	3 (truck; saloon and SUV; bus)	2346	SVM	95.36%
[38]	side of the road	5 (sedan; van; truck; bus; non-vehicle)	412	DTW + VQ	94.6%
[39]	side of the road	7 (motorcycle; car; SUV; truck; crane; medium truck; bus)	6042	CNN	97.09%
[1]	middle of the lane	5 (passenger vehicle; SUV; van; pickup; bus)	37	Direct Hill-Pattern Matching	63%
[40]	middle of the lane	2 (car; bus)	542	CT	97.0% on training data, 88.9% on validation data
[25]	middle of the lane	4 (car; minibus; bus; truck)	100	CT	95%
[27]	middle of the lane	4 (sedan and SUV; van and seven-seat; light and medium truck; heavy truck and semi-trailer)	4507	CT	80.55% on validation data
[41]	middle of the lane	4 (sedan; SUV and van; bus; truck)	115	SVM	87%
[42]	middle of the lane (1 board with 2 sensors)	4 (motorcycle; car; van; pickup)	130	CT	81.69%
[43]	middle of the lane (1 board with 2 sensors)	3 (bus; small and medium truck; large truck)	460	SVM	92.8%
[32]	atop of the roadway	3 (passenger car, 2-axle single-unit vehicles; 2-axle and 3-axle single-unit trucks; 5-axle single-trailer trucks)	1985	SVM	86.85%

**Table 2:** Used feature types in related works.

Related Work	Data Type	Features
[10]	*X*	concavity area, convexity area, the angle of concave part, the angle of convex part of the waveform
[26]	*X, Y*	vehicle signal duration, signal energy, average energy, ratio of positive and negative energy
[36]	*X, Y, Z, F*	position of the maximum, position of the minimum, detection length, peak-to-peak value, mean value, standard deviation, number of extremes, the sign of the first extreme, the number of zero-crossings, energy of the detected signal, average energy, ratio of the energy of the signals on the sensor’s axis to the energy of the *F* signal, first non-zero samples of the frequency spectrum
[1]	*X, Z*	Hill-patterns
[40]	*F*	number of peaks, maximum peak time ratio, minimum trough time ratio, mean value, the standard deviation, the maximum peak amplitude, the minimum trough amplitude, maximum peak/trough amplitude ratio
[25]	*F*	magnetic signature length
[27]	*Z*	statistical features: magnetic length, mean, variance, maximum and minimum, position of the maximum and minimum, number of local maxima and minima, crossing mean counts; energy features: energy, mean energy; short-term features: mean, variance and energy computed in intra-frames of the detection window
[42]	*Z*	signal length, relative vehicle length, Hill-pattern peaks, three differential energy parameters
[43]	*F*	structural features: number of local maxima, local minima, extreme points, and negative local minima, relative time of minimum and maximum, penultimate minimum/minimum; spectrum features: highest spectrum power and the corresponding frequency; numerical features: maximum value, minimum value, sum of value, average value, max value/min value, max average value/average value, standard deviation, on-time speed
[37]	*X, Y, Z*	maximum, range, relative position of maximum, relative position of minimum, mean, ratio of positive and negative energy, number of local maxima, number of local minima, variance, approximate entropy, crossing mean counts, average energy
[38]	*F*	MFCC, energy
[41]	*X, Y, Z*	HOG features using image processing
[39]	*X, Y, Z*	224 × 244 grayscale images containing the waveforms
[32]	*F*	FFT + PCA

**Table 3:** Hyperparameters of the MLP structure.

Hyperparameter	Value
number of layers	3 (1 input, 1 hidden, 1 output)
number of neurons in the input layer	the size of the feature vector
number of hidden layer neurons	optimal number given by the best results
transfer function in the hidden layer	tangent sigmoid
number of neurons in the output layer	the number of defined classes
transfer function in the output layer	linear

**Table 4:** Used vehicle classes.

Class Number	Vehicle Types	Number of Axles
1	motorcycle	2
2	car	2
3	car with trailer	2 + 1
4	van, mini bus	2
5	truck	2–3
6	truck with trailer	2–3 + 2–3
7	tractor trailer	2 + 3
8	bus	2
9	articulated bus	3

**Table 5:** Hyperparameters of the MLP training process.

Hyperparameter	Value
training function	Levenberg-Marquardt backpropagation
performance function	mean squared error (MSE)
maximum number of epochs to train	5000
performance goal	0
maximum validation failures	15
minimum performance gradient	10^−7^
maximum time to train in seconds	inf

**Table 6:** Feature numbers and average recognition efficiencies (%) on training and validation data for different setups.

Used Axes											
Feature Number											
Efficiency on Training Data						Efficiency on Validation Data					
*X*, *Y*, *Z*		*X*, *Y*, *Z*, DL		*X*		*X*, DL		*Z*		*Z*, DL	
42		43		14		15		14		15	
82.11	67.69	81.50	66.58	78.83	71.33	80.11	72.22	77.92	71.24	81.36	72.36
*X*, *Z*		*X*, *Z*, DL		*X_abs_*, *Y_abs_*, *Z_abs_*		*X_abs_*, *Y_abs_*, *Z_abs_*, DL		*X_abs_*		*X_abs_*, DL	
28		29		27		28		9		10	
81.61	70.13	81.97	71.64	81.00	71.20	81.81	69.73	73.86	68.18	76.14	70.04
*Z_abs_*		*Z_abs_*, DL		*X_abs_*, *Z_abs_*		*X_abs_*, *Z_abs_*, DL		*XY_O_*, *XZ_O_*, *YZ_O_*, *XYZ_O_*		*XY_O_*, *XZ_O_*, *YZ_O_*, *XYZ_O_*, DL	
9		10		18		19		56		57	
74.31	69.07	75.56	69.82	78.97	69.96	80.08	71.38	82.20	70.18	82.81	68.18
*XZ_O_*		*XZ_O_*, DL		*XY_O_*, *XZ_O_*, *YZ_O_*		*XY_O_*, *XZ_O_*, *YZ_O_*, DL		*XYZ_O_*		*XYZ_O_*, DL	
14		15		42		43		14		15	
80.75	73.73	80.14	74.44	81.03	69.82	84.06	71.51	77.97	72.76	81.39	74.67
*XY_A_*, *XZ_A_*, *YZ_A_*		*XY_A_*, *XZ_A_*, *YZ_A_*, DL		*XZ_A_*		*XZ_A_*, DL		*XY_C_*, *XZ_C_*, *YZ_C_*, *XYZ_C_*		*XY_C_*, *XZ_C_*, *YZ_C_*, *XYZ_C_*, DL	
42		43		14		15		36		37	
79.00	66.53	79.97	65.78	80.92	71.20	79.69	71.87	79.08	66.36	81.75	69.11
*XZ_C_*		*XZ_C_*, DL		*XY_C_*, *XZ_C_*, *YZ_C_*		*XY_C_*, *XZ_C_*, *YZ_C_*, DL		*XYZ_C_*		*XYZ_C_*, DL	
9		10		27		28		9		10	
72.78	68.49	76.53	70.53	80.83	68.36	79.28	68.27	73.61	68.93	75.69	71.38

**Table 7:** Average misclassification rates (%) on validation data using the XZ_O_-based features with (upper row) and without (lower row) using the detection length feature.

		Output Class									Sum
		1	2	3	4	5	6	7	8	9	
**Target class**	**1**		1.6		0.4	1.2					3.2
			2.8		0.4						3.2
	**2**	2.0		2.8	4.8	0.4					10.0
		2.0		2.4	6.8	1.2					12.4
	**3**		4.8		7.2	10.8		1.2	1.2		25.2
			7.2		10.0	10.8	0.4	0.4	1.2		30.0
	**4**	0.8	10.4	7.6		24.0		0.4	1.6		44.8
		0.8	17.6	8.0		24.0			1.6		52.0
	**5**		0.8	6.0	10.8		0.8	2.8	12.4	1.2	34.8
			1.2	4.8	8.4		0.8	2.8	18.0	0.4	36.4
	**6**			0.8	0.4	2.4		16.8	4.4	13.2	38.0
				2.0		1.6		21.6	4.0	11.6	40.8
	**7**			1.6	0.4	2.4	13.6		12.8	8.4	39.2
				0.4		2.0	16.4		10.8	9.6	39.2
	**8**				0.8	3.2	1.2	3.6		2.4	11.2
					0.4	4.0	0.8	4.0		4.4	13.6
	**9**					0.4	8.8	2.8	11.6		23.6
					0.4		6.8	4.0	5.6		16.8

**Table 8:** Required number of hidden layer neurons for reaching 97% convergence on the 36 datasets.

**Hidden Layer Neuron Number**	5	10	15	20	25	30
**Percentage of Convergence For Training Data**	0.00%	2.78%	19.44%	30.56%	38.89%	8.33%
**Percentage of Convergence For Validation Data**	0.00%	58.33%	30.56%	8.33%	2.78%	0.00%

## Data Availability

Not applicable.
